# Evaluation of Antibody Response in Symptomatic and Asymptomatic COVID-19 Patients and Diagnostic Assessment of New IgM/IgG ELISA Kits

**DOI:** 10.3390/pathogens10020161

**Published:** 2021-02-03

**Authors:** Hadeel T. Al-Jighefee, Hadi M. Yassine, Maryam A. Al-Nesf, Ali A. Hssain, Sara Taleb, Ahmed S. Mohamed, Hassen Maatoug, Mohamed Mohamedali, Gheyath K. Nasrallah

**Affiliations:** 1 Biomedical Research Center, Qatar University, Doha 2713, Qatar; hadeel.mohammed@qu.edu.qa (H.T.A.-J.); hyassine@qu.edu.qa (H.M.Y.); 2 Department of Biomedical Science, College of Health Sciences, Member of QU Health, Qatar University, Doha 2713, Qatar; 3 Adult Allergy and Immunology Section, Department of Medicine, Hamad Medical Corporation, Doha 2713, Qatar; Mariamali@hamad.qa; 4 Medical Intensive Care Unit, Hamad Medical Corporation, Doha 2713, Qatar; AHssain@hamad.qa; 5 Division of Genomics and Translational Biomedicine, College of Health and Life Sciences, Hamad Bin Khalifa University, Doha 2713, Qatar; staleb@hbku.edu.qa; 6 Criticl Care Nursing Department, Hazm Mebaireek General Hospital (HMGH), Hamad Medical Corporation, Doha 2713, Qatar; amohamed@hamad.qa; 7 Nursing Deaprtment, Hamad Medical Corporation, Doha 2713, Qatar; hmaatoug@hamad.qa; 8 Department of Medicine, Hazm Mebaireek General Hospital (HMGH), Hamad Medical Corporation, Doha 2713, Qatar; MMohamedali@hamad.qa

**Keywords:** COVID-19, SARS-CoV-2, serology, IgG, IgM, ELISA, sensitivity, specificity, symptomatic, asymptomatic

## Abstract

This study aims to study the immune response and evaluate the performances of four new IgM and five IgG enzyme-linked immunosorbent assay (ELISA) kits for detecting anti-severe acute respiratory syndrome coronavirus 2 (SARS-CoV-2) antibodies against different antigens in symptomatic and asymptomatic coronavirus disease 2019 (COVID-19) patients. A total of 291 samples collected from symptomatic and asymptomatic RT–PCR-confirmed patients were used to evaluate the ELISA kits’ performance (EDI, AnshLabs, DiaPro, NovaLisa, and Lionex). The sensitivity was measured at three different time-intervals post symptoms onset or positive SARS-CoV-2 RT–PCR test (≤14, 14–30, >30 days). The specificity was investigated using 119 pre-pandemic serum samples. The sensitivity of all IgM kits gradually decreased with time, ranging from 48.7% (EDI)–66.4% (Lionex) at ≤14 days, 29.1% (NovaLisa)–61.8% (Lionex) at 14–30 days, and 6.0% (AnshLabs)–47.9% (Lionex) at >30 days. The sensitivity of IgG kits increased with time, peaking in the latest interval (>30 days) at 96.6% (Lionex). Specificity of IgM ranged from 88.2% (Lionex)–99.2% (EDI), while IgG ranged from 75.6% (DiaPro)–98.3% (Lionex). Among all RT–PCR-positive patients, 23 samples (7.9%) were seronegative by all IgG kits, of which only seven samples (30.4%) had detectable IgM antibodies. IgM assays have variable and low sensitivity, thus considered a poor marker for COVID-19 diagnosis. IgG assays can miss at least 8% of RT–PCR-positive cases.

## 1. Introduction

The current coronavirus disease 2019 (COVID-19) pandemic has imposed an unpreceded challenge on the health and economy of millions of people. As of 22 January 2021, the global number of confirmed COVID-19 cases have exceeded 96 million cases, with more than 2.07 million known fatalities. Up to date, there is no sufficiently effective antiviral drug to treat COVID-19. Therefore, the development of accurate and reliable diagnostic serological assays that can be readily applicable is crucial [1,2]. These assays should provide guidance to determine the seroprevalence of antibodies against the severe acute respiratory syndrome coronavirus 2 (SARS-CoV-2) at the individual and community levels, identify immune presumptive protected persons who can serve as potential plasma donors, and for vaccine development [1]. In addition, serology assays are expected to play a critical role in testing the currently approved vaccines’ efficiency by measuring the level of produced antibodies, determining their durability, and identifying thresholds of protection [3,4]. This is particularly important since it is still unknown how long immunity against SARS-CoV-2 lasts after vaccination [3]. Due to the lack of sensitive and specific serological assays early in the COVID-19 pandemic, there was a delay in the precise estimation of the burden of infection; and hence, the proper implementation of public health measures to control viral spread [5]. Currently, the diagnosis of COVID-19 is based on patient history, laboratory testing, and chest X-ray examination. The reference method for SARS-CoV-2 detection is nucleic acid testing (NAT) of respiratory specimens. However, this method is low throughput, time-consuming, should be performed by professional technicians and requires additional sampling for an accurate diagnosis. Thus, NAT may not be the best choice for large-scale screening of populations infected with SARS-CoV-2 [6].

The detection of serum-specific IgM and IgG is routinely used in clinical laboratories to provide insights regarding the virus infection time course. IgM antibodies are produced as the first line of defense during infection, where these antibodies are used to evaluate the acute phase of infection as they indicate recent exposure. In contrast, IgG antibodies are generated afterward to provide long-term immunity and immunological memory [7]. As has been shown in several studies, the seroconversion of IgG and IgM occurs about one to two weeks after disease onset, and the levels of IgM significantly drop while IgG persists for a longer period of time [8,9]. Hence, IgM and IgG antibodies, when captured within the correct timeframe after disease onset, can add value to the diagnosis and treatment of COVID-19. Understanding the antibody kinetics over time is essential to distinguish thresholds of immunity, especially since it is still unknown how long the immunity to this novel coronavirus might last [1,5]. Although RT–PCR remains the reference method for identifying acute infection, as the SARS-CoV-2 pandemic continues to spread, serological testing has become essential to understand the pandemics’ past and predict its future [5].

Recent seroprevalence studies have strongly suggested that COVID-19 cases, especially the asymptomatic, are greatly underestimated. Studies performed on large populations have shown a 1.2–12.9% SARS-COV-2 incidence rate of asymptomatic cases, which significantly contributed to the disease transmission [10,11,12,13]. Early identification and quarantining of these individuals are urgently needed to better control the COVID-19 pandemic. This study evaluated the antibody immune response in symptomatic and asymptomatic RT–PCR-confirmed COVID-19 patients using different CE-marked IgM and IgG ELISA kits coated with different SARS-CoV-2 antigens. IgM and IgG antibodies were detected in samples collected at three different time-intervals post symptoms onset or positive SARS-CoV-2 RT–PCR test (≤14, 14–30, >30 days). Specificity was investigated using pre-pandemic control samples with positive antibodies against other coronaviruses, non-CoV respiratory viruses, non-respiratory viruses, and nuclear antigens. A strength of this study is that it evaluated the antibody response using a large sample size from both symptomatic and asymptomatic patients of a very diverse population in Qatar, where 89% of the total population are expatriates from over 150 countries [14,15,16,17].

## 2. Materials and Methods

### 2.1. Study Design, Ethical Compliance, and Sample Collection

The performances of four IgM (EDI, NovaLisa, AnshLabs, and Lionex) and five IgG ELISA kits (EDI, NovaLisa, AnshLabs, DiaPro, and Lionex) for detecting anti-SARS-CoV-2 antibodies were evaluated. The performance was assessed using samples collected from symptomatic and asymptomatic SARS-CoV-2 RT–PCR-confirmed patients. A panel of 119 pre-pandemic serum samples collected from healthy blood donors was selected as the negative control group. The IRB approvals for this study were obtained from Hamad Medical Corporation (HMC-IRB# MRC-01-20-145, HMC-IRB# MRC-05-003, and HMC-IRB# MRC-05-007) and Qatar University (QU-IRB # QU-IRB 804-E/17).

### 2.2. Serum Samples

A total of 291 serum samples from confirmed COVID-19 patients were selected, including symptomatic (*n* = 147) and asymptomatic (*n* = 119) patients. Samples were classified based on the day of collection post symptoms onset (DPSO) for symptomatic patients or days post-diagnosis (DPD) with positive SARS-CoV-2 RT-PCR test for asymptomatic individuals: ≤14 (*n* = 119), 14–30 (*n* = 55), >30 days (*n* = 117). Nasopharyngeal swab specimens from all patients were tested for SARS-CoV-2 using the Superscript III One-Step RT-PCR reaction mix with PlatinumR Taq DNA polymerase (ThermoFisher, Waltham, Massachusetts, Mass, USA). Each sample was tested by three PCRs: the screening assay for the envelope (E) gene and two confirmatory assays targeting the RNA dependent RNA polymerase (RdRp) gene, all performed as recommended by Corman, V.M. et al. [18]. Quant Studio 6 Flex real-time PCR System was used, and cycle threshold (CT) values below 32 were considered positive. For the negative control group, serum samples collected from healthy blood donors before 2019 were selected. Details about the collection, transport, and storage methods of the control samples were described in previous studies [19,20,21,22,23,24,25,26]. The demographic and clinical characteristics of COVID-19 patients and the control group are shown in Table 1. The median age of the control group was 36 years (interquartile range: IQR = 15). Most of the control group samples were within the 31–60 years age group (*n* = 82, 68.9%). In the control group, there was an equal representation of females (*n* = 57, 49.6%) to males (*n* = 59, 51.3%). In the COVID-19 patient group, the median age was 43 years (IQR = 21), and most patients were within the age group 31–60 years (*n* = 195, 67.0%). There was a higher proportion of COVID-19 male patients (*n* = 242, 83.2%) compared to the females (*n* = 33, 11.3%). Among COVID-19 patients, there was an approximately equal distribution of symptomatic (*n* = 147, 55.9%) and asymptomatic patients (*n* = 116, 44.1%).

### 2.3. SARS-CoV-2 Antibodies Detection Using Enzyme-Linked Immunosorbent Assay (ELISA)

Commercial ELISA kits from different companies targeting IgM and IgG antibodies against recombinant nucleocapsid protein (NP) alone, both nucleocapsid and spike proteins (NP and SP), and S1 protein of SARS-CoV-2 were evaluated. The kits are (i) Epitope Diagnostic (EDI™) novel coronavirus COVID-19 IgM/IgG (ref. no. KT-1033 and KT-1032,California, CA, USA), which detects anti-NP antibodies (ii) AnshLabs SARS-CoV-2 IgM/IgG (ref. no. AL-1002-I and AL-1001-I, Texas, TX, USA), which detects both anti-NP and -SP antibodies (iii) Diagnostic Bioprobes (DiaPro) COVID-19 IgG (COV19G.CE, Milan, Italy), which also detects anti-NP and -SP antibodies (iv) NovaLisa^®^ SARS-CoV-2 IgM/IgG (ref. no. COVM0940 and COVG0940, Hessen, Germany), which detects anti-NP antibodies, and (v) Lionex COVID-19 IgM/IgG ELISA (ref. no. LIO-COV19-IgM and LIO-COV19-IgG, Braunschweig, Germany), which detects anti-S1 antibodies. Further details about the kit’s characteristics are shown in Table 2. All tests were carried out manually according to the manufacturers’ instructions.

### 2.4. Statistical Analysis

Using RT–PCR as the reference standard, sensitivity, specificity, overall agreement, positive predictive value, negative predictive value, and Cohen’s kappa statistic were calculated to assess the performance of each assay. Borderline results were considered positives [27,28]. Cohen’s kappa statistic was used to estimate the level of agreement between every two kits. Ranging between 0 and 1, a kappa value of ≤0.40 indicating poor agreement, a value between 0.40–0.75 indicating fair/good agreement, and a value ≥0.75 indicating excellent agreement [29]. The level of significance was set at 5%, and the 95% confidence interval (CI) was estimated for each measure. Statistical analyses were performed using Microsoft Excel 2016 and GraphPad Prism Version 9.0 (GraphPad, California, CA, USA). Data are presented as mean ± SEM. Statistical analyses were performed using one-way analysis of variance (ANOVA) and the Kruskal–Wallis test, followed by Dunn’s post hoc tests. Chi-squared was used to evaluate the significance of the sensitivity for each kit. Significant differences were represented as: (*) for *p* < 0.05; (**) for *p* < 0.01; (***) for *p* < 0.001.

## 3. Results

### 3.1. Diagnostic Assessment of IgM ELISA Kits

#### 3.1.1. Assays Assessment at Different Time Intervals from Sample Collection

**Figure 1:**
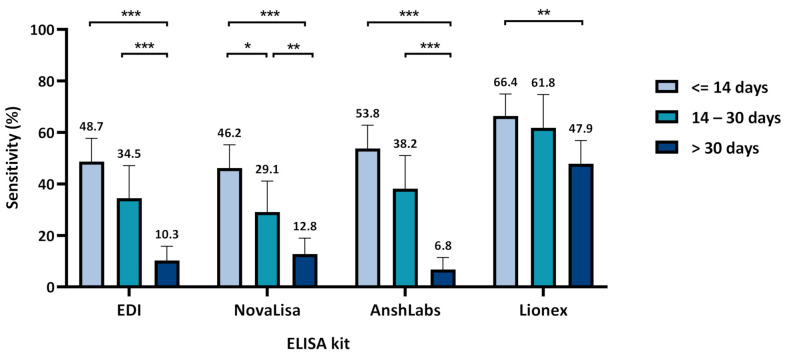
Assays sensitivity according to time of sample collection after symptoms onset or positive severe acute respiratory syndrome coronavirus 2 (SARS-CoV-2) RT-PCR for both symptomatic and asymptomatic patients. Chi-squared test was used to detect the presence of a statistically significant difference in the sensitivity between the time-intervals for each assay, * *p* < 0.05, ** *p* < 0.01, *** *p* < 0.001.

Figure 1 and Table S1 summarize the diagnostic assessment of each IgM ELISA kit at the three time-intervals of sampling post symptoms onset or positive SARS-CoV-2 RT–PCR test (≤14, 14–30, >30 days). The sensitivity of the four IgM ELISA significantly decreased with time, ranging from 48.7% (95% CI: 39.8–57.7) for EDI to 66.4% (95% CI: 57.9–74.9) for Lionex at the first time-interval (≤14 days). The sensitivity significantly decreased in the second time-interval (14–30 days) for NovaLisa (29.1%) but remained relatively high in Lionex (61.8%). The highest overall sensitivity, agreement with RT–PCR, and the negative predictive value was scored by Lionex at 58.4% (95% CI: 52.8–64.1), 67.1% (95% CI: 62.5–71.6), and 46.5% (95% CI: 41.6–51.3), respectively (Table S1).

Since most of the asymptomatic samples were collected at the third time-interval (>30 days), the sensitivity at the three time-points was re-calculated using the symptomatic samples alone for more accurate estimation. As shown in Figure S1, the sensitivity increased by all ELISA kits in the first time-interval (≤14 days), ranging from 62.4% (95% CI: 52.1–72.7) for NovaLisa to 85.9% (95% CI: 78.5–93.3) for Lionex. The sensitivity was also relatively high in the second time-interval (14–30 days), ranging from 54.2% (95% CI: 34.2–74.1) for NovaLisa to 75.0% (95% CI: 57.7–92.3) for Lionex. However, the sensitivity significantly decreased in the third time-interval for all kits, ranging from 2.6% (95% CI: −2.5–7.7) for NovaLisa to 52.6% (95% CI: 36.8–68.5) for Lionex.

#### 3.1.2. Assays Assessment in Symptomatic and Asymptomatic COVID-19 Patients

**Figure 2:**
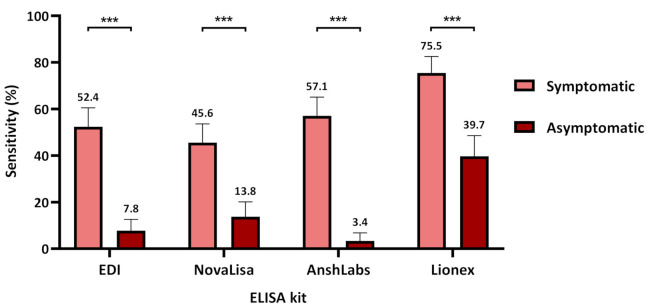
Assays sensitivity according to coronavirus disease 2019 (COVID-19) patient classification (symptomatic or asymptomatic). Chi-squared was used to calculate the significance between the sensitivities in symptomatic and asymptomatic patients for each kit, *** *p* < 0.001.

Figure 2 and Table S1 summarize the diagnostic assessment of each IgM ELISA in symptomatic and asymptomatic COVID-19 patients. The sensitivity was significantly higher in the symptomatic COVID-19 patients by all kits where Lionex demonstrated the highest sensitivity at 75.5% (95% CI: 68.6–82.5), followed by AnshLabs at 57.1% (95% CI: 49.1–65.1), then EDI at 52.4% (95% CI: 44.3–60.5), and finally NovaLisa at 45.6% (95% CI: 37.5–53.6). The sensitivity in the asymptomatic COVID-19 patients ranged from 3.4% (95% CI: 0.13–6.8) to 39.7% (96% CI: 30.8–48.6) for AnshLabs and Lionex, respectively.

**Figure 3:**
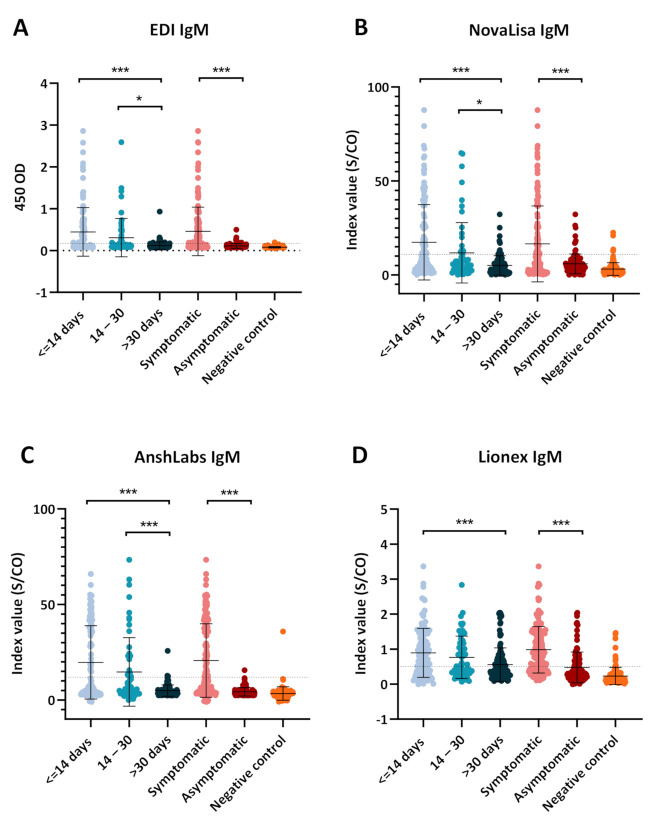
Dot plot distribution of the IgM ELISA index values according to the different time points of sampling (≤14, 14–30, >30 days) and coronavirus disease 2019 (COVID-19) patient classification (symptomatic or asymptomatic). Each dot plot represents the index values obtained with each serological assay: (**A**) EDI™, (**B**) NovaLisa, (**C**) AnshLabs, and (**D**) Lionex. Results are expressed as a ratio of the sample signal to the cutoff for all tests except the EDI™ assay, which is expressed in optical density. One-way analysis of variance (ANOVA) was used to compare the differences between groups, * *p* < 0.05, *** *p* < 0.001.

The dot plot distribution of the results obtained from each IgM ELISA kit is shown in Figure 3. All kits showed a significant drop in the index value with time, where the highest values were obtained during the first time-interval (≤14 days). Symptomatic COVID-19 patients also demonstrated significantly higher index values compared to the asymptomatic patients by all ELISA kits. EDI IgM ELISA demonstrated the clearest separation of known positive and known negative samples (Figure 3A). NovaLisa also showed the clearest separation of the different time-intervals of sample collection post symptoms onset or positive SARS-CoV-2 RT–PCR test (Figure 3B).

### 3.2. Diagnostic Assessment of IgG ELISA Kits

#### 3.2.1. Assays Assessment at Different Time Intervals of Sample Collection

**Figure 4:**
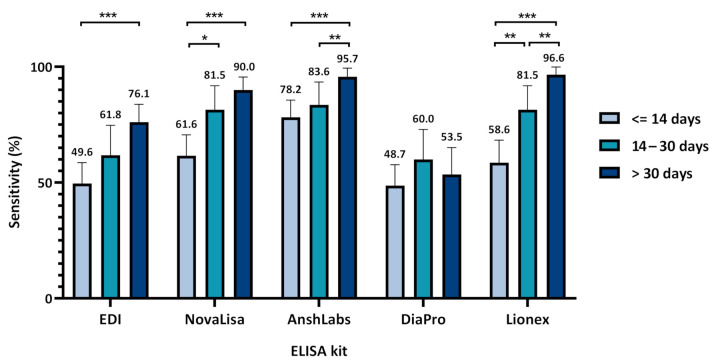
Assays sensitivity according to time of sampling after symptoms onset or positive severe acute respiratory syndrome coronavirus 2 (SARS-CoV-2) RT–PCR test. Chi-squared test was used to detect the presence of a statistically significant difference in the sensitivity between the time-intervals for each assay, * *p* < 0.05, ** *p* < 0.01, *** *p* < 0.001.

Figure 4 and Table S1 summarize the diagnostic assessment of the five IgG ELISA kits. The lowest sensitivities were observed in samples collected at the first time-interval (≤14 days) by all IgG ELISA kits, ranging from 48.7% (95% CI: 39.8–57.7) for DiaPro to 78.2% (95% CI: 70.7–85.6) for AnshLabs. The sensitivities of all kits increased after the second week of sample collection (14–30 days), peaking at 83.6% (95% CI: 73.9–93.4) in AnshLabs. The highest sensitivities were observed in samples collected after one month (>30 days) by all kits, except DiaPro, which dropped down from 60.0% (95% CI: 47.1–72.9) at the second time-interval to 53.5% (95% CI: 41.9–65.1). Both AnshLabs and Lionex showed sensitivities above 95% at the third time point (95.7% and 96.6%, respectively). The highest overall sensitivity, agreement with RT–PCR, and the negative predictive value was scored by AnshLabs at 86.3% (95% CI: 82.3–90.2), 85.6 (95% CI: 82.2–89.0), and 71.4% (95% CI: 67.1–75.8), respectively (Table S1).

#### 3.2.2. Assays Assessment in Symptomatic and Asymptomatic COVID-19 Patients

**Figure 5:**
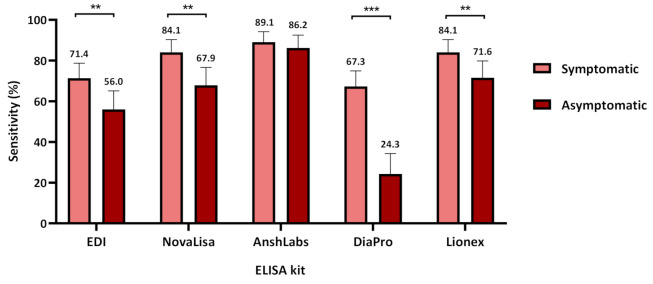
Assays sensitivity according to coronavirus disease 2019 COVID-19 patient classification (symptomatic or asymptomatic). Chi-squared was used to calculate the significance between the sensitivities in symptomatic and asymptomatic patients for each assay, ** *p* < 0.01, *** *p* < 0.001.

Figure 5 shows the sensitivity assessment of each IgG ELISA kit in symptomatic and asymptomatic COVID-19 patients. The sensitivity was significantly higher in symptomatic patients by all kits where AnshLabs showed the highest sensitivity at 89.1% (95% CI: 84.1–94.2), followed by both NovaLisa and Lionex at 84.1% (95% CI: 77.9–90.3), then EDI at 71.4% (95% CI: 64.1–78.7), and DiaPro at 67.3% (95% CI: 59.8–74.9). The sensitivity in asymptomatic COVID-19 patients ranged from 24.3% (95% CI: 14.2–34.3) to 86.2% (96% CI: 79.9–92.5) for DiaPro and AnshLabs, respectively.

**Figure 6:**
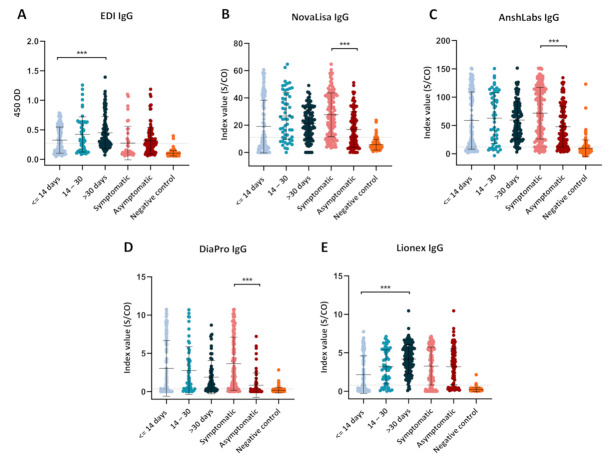
Dot plot distribution of the IgG ELISA index values according to the different time points of sampling (≤14, 14–30, >30 days) and coronavirus disease 2019 (COVID-19) patient classification (symptomatic or asymptomatic). Each dot plot represents the index values obtained with each serological assay: (**A**) EDI™, (**B**) NovaLisa, (**C**) AnshLabs, (**D**) DiaPro, and (**E**) Lionex. Results are expressed as a ratio of the sample signal to the cutoff for all tests except the EDI™ assay, which is expressed in optical density. One-way analysis of variance (ANOVA) was used to compare the differences between groups, *** *p* < 0.001.

The dot plot distribution of the results obtained from each IgG ELISA kit is shown in Figure 6. Only EDI and Lionex showed a significant increase in the index value between the first (≤14 days) and third (>30 days) time-intervals (Figure 6A,E). NovaLisa, DiaPro, and AnshLabs demonstrated significantly higher index values in symptomatic COVID-19 patients compared to the asymptomatic patients (Figure 6B–D).

### 3.3. Specificity According to the Negative Control Subgroups for IgM and IgG ELISA

Table 3 summarizes the overall specificity and specificity for each control subgroup of the IgM and IgG ELISA kits. The overall specificities of the four IgM ELISA kits ranged from 88.2% (96% CI: 82.4–92.0) to 99.2% (95% CI: 97.5–100) in the pre-COVID-19 samples. EDI showed the highest specificity in all subgroups and cross-reacted with only one sample positive for antibodies against influenza (98.5%).

The overall specificity of the IgG ELISA was also good, ranging from 75.6% (67.9–83.3) to 98.3% (95% CI: 96.0–100), where three kits demonstrated an overall specificity above 90% (EDI: 98.3%, NovaLisa: 96.5%, and Lionex: 97.5%). DiaPro had the lowest specificity among the IgG ELISA kits, with an overall specificity of 75. 6% (95% CI: 67.9–83.3).

The EDI ELISA showed the best specificity in detecting both IgG and IgM antibodies against SARS-CoV-2 (98.3% and 99.2%, respectively). Lionex, however, showed very high specificity in detecting IgG antibodies (97.5%) but low specificity in detecting IgM antibodies (88.2%).

### 3.4. Agreement of IgM and IgG ELISA Kit

**Figure 7:**
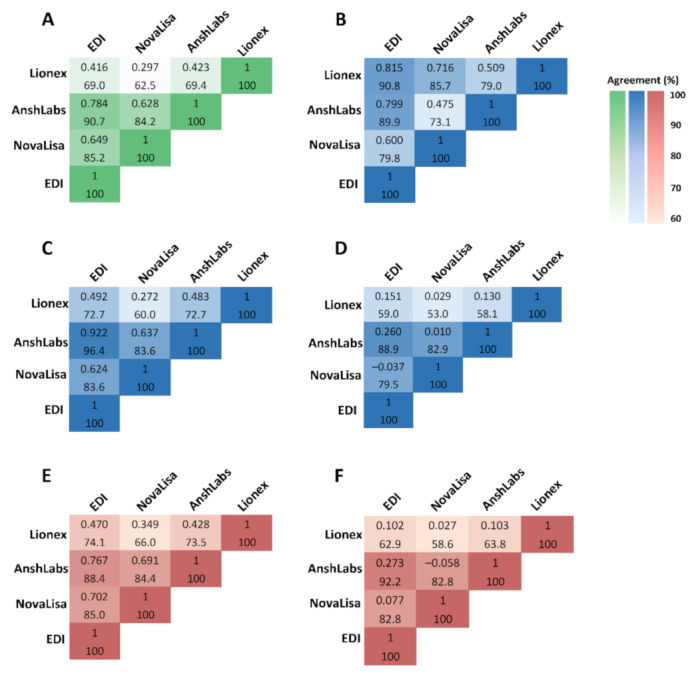
Concordance assessment for the overall agreement and kappa (k) among all IgM ELISA tests. (**A**) Overall agreement, (**B**) agreement in samples collected ≤14 DPSO/DPD, (**C**) agreement in samples collected 14–30 DPSO/DPD, (**D**) agreement in samples collected >30 DPSO/DPD, (**E**) agreement in samples collected from symptomatic coronavirus disease 2019 (COVID-19) patients, (**F**) agreement in samples collected from asymptomatic COVID-19 patients.

As shown in Figure 7A, the pairwise comparison between the IgM ELISA kits showed that EDI and NovaLisa had the best overall agreement [90.7% (95% CI: 87.4–94.1)] and the kappa index indicated an excellent agreement (k = 0.784). AnshLabs and EDI along with AnshLabs and NovaLisa also showed a very good agreement (85.2% (95% CI: 81.1–89.3), k = 0.649 and 84.2% (95% CI: 80.0–88.4), k = 628, respectively).

In the first time-interval (≤14 days), EDI and Lionex demonstrated the best agreement at 90.8% (95% CI: 85.6–96.0) and a kappa index of 0.815 (Figure 7B). However, in the second time-interval (14–30 days), EDI and AnshLabs showed the best agreement at 96.4% (95% CI: 91.4–100) with a kappa index of 0.922 (Figure 7C). In the third time-interval (>30 days), EDI and AnshLabs also showed the best agreement at 88.9% (95% CI: 83.2–94.6) and a kappa index of 0.260 (Figure 7D).

In the symptomatic COVID-19 patient group, EDI and AnshLabs had the best agreement at 88.4% = (95% CI: 83.3–93.6) and a kappa index of 0.767, indicating an excellent agreement (Figure 7E). EDI and AnshLabs also showed the best agreement in the asymptomatic COVID-19 patient group at 92.2% (95% CI: 87.4–97.1), but the kappa index was low at 0.273 (Figure 7F).

**Figure 8:**
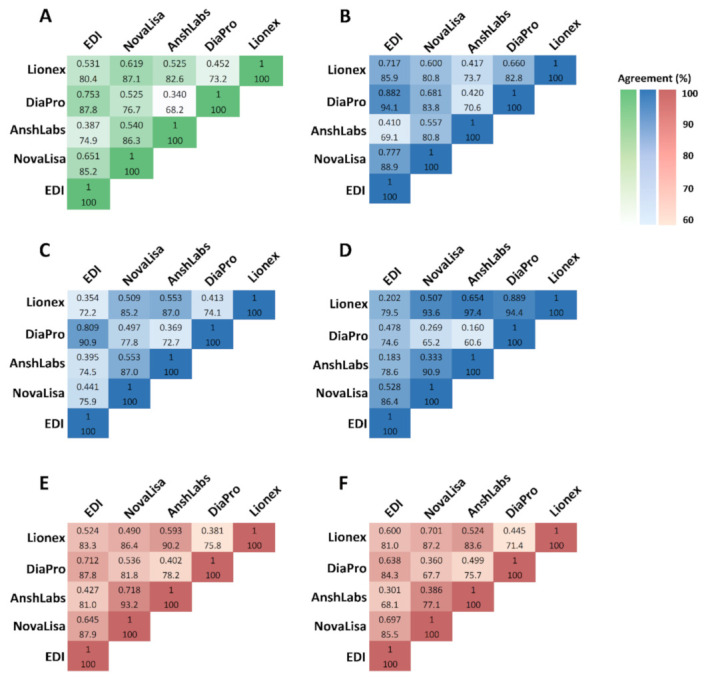
Concordance assessment for the overall agreement and kappa (k) among all IgG ELISA tests. (**A**) Overall agreement, (**B**) agreement in samples collected ≤14 DPSO/DPD, (**C**) agreement in samples collected 14–30 DPSO/DPD, (**D**) agreement in samples collected >30 DPSO/DPD, (**E**) agreement in samples collected from symptomatic coronavirus disease 2019 (COVID-19) patients, (**F**) agreement in samples collected from asymptomatic COVID-19 patients.

Figure 8 summarizes the pairwise comparison between the five IgG ELISA kits. EDI and DiaPro showed the best overall agreement at 87.8% (95% CI: 83.7–91.9) and a kappa index of 0.753, indicating an excellent agreement (Figure 8A). NovaLisa and Lionex, along with NovaLisa and AnshLabs also showed a very good agreement 87.1% (95% CI: 83.0–91.1), k = 0.619 and 86.3% (95% CI: 82.2–90.5), k = 540, respectively.

In the first time-interval (≤14 days), EDI and DiaPro demonstrated the best percent agreement at 94.1% (95% CI: 89.9–98.3) and had an excellent kappa index at 0.882 (Figure 8B). In the second time-interval (14–30 days), EDI and DiaPro also had the best agreement at 90.9% (95% CI: 83.3–98.5) with a kappa index of 0.809 (Figure 8C). In the third time-interval (>30 days), Lionex showed the best agreement with NovaLisa, DiaPro, and AnshLabs at 93.6% (95% CI: 89.1–98.2), 97.4% (95% CI: 94.6–100), and 94.4% (95% CI: 89.0–99.7), respectively (Figure 8D).

In the symptomatic COVID-19 patient group, NovaLisa and AnshLabs had the best agreement at 93.2% (95% CI: 88.9–97.5) and a kappa index of 0.718, indicating a very good agreement (Figure 8E). In the asymptomatic COVID-19 patient group, NovaLisa and Lionex had the best agreement at 87.2% (95% CI: 80.9–93.4) with a kappa index of 0.701 (Figure 8F).

### 3.5. Negative Samples by All IgG ELISA Kits

Among the 291 samples collected from positive SARS-CoV-2 RT–PCR patients, 23 samples (7.9%) were seronegative by all IgG ELISA kits despite testing with multiple assays targeting different antigens (Table 4). Among these samples, 17 samples (73.9%; 17/23) were collected during the first time-interval (≤14 days), three samples (13.0%, 3/23) were collected during the second time-interval (14–30 days), and three samples (13.0%, 3/23) were collected after one month. Eleven samples (47.8%, 11/23) were collected from symptomatic COVID-19 patients, and 12 samples (52.2%, 12/23) were from asymptomatic patients. Only seven samples (30.4%) had detectable IgM antibodies by at least one of the four IgM ELISA kits.

## 4. Discussion

Since the start of the COVID-19 pandemic, numerous commercial serological assays have been developed and approved. This study describes the test performance of four new IgM and five IgG ELISA kits targeting antibodies against different SARS-CoV-2 antigens. A panel of 291 samples collected from RT–PCR confirmed COVID-19 patients, and 119 pre-pandemic serum samples were used to evaluate the performance of the assays. The sensitivity was evaluated at different time-intervals post symptoms onset or positive SARS-CoV-2 RT–PCR test: ≤14, 14–30, >30 days. In addition, we compared the test performance of the IgM and IgG ELISA kits.

Previous studies on SARS-CoV-2 showed that IgM antibodies could be detected as early as three days post-infection, providing the first line of humoral immunity defense, while high-affinity IgG antibodies are produced after seven days [30,31]. In this study, the sensitivity for IgM antibody detection decreased gradually with time using all ELISA kits. The highest sensitivity was obtained during the first two weeks of sample collection (Figure 1 and Figure S1). Three IgM ELISA kits that either target the nucleocapsid protein (NP) or both the nucleocapsid and spike protein (SP) showed relatively lower sensitivity than Lionex, which solely targets the SP of SARS-CoV-2. The sensitivities of all IgG ELISA kits increased proportionally to the elapsed time from symptoms onset or positive RT–PCR test, peaking one month after sample collection, except for DiaPro. AnshLabs IgG ELISA attained the highest sensitivity within the first two weeks of sampling (78.2%), while Lionex achieved the highest sensitivity after one month (96.6%). This indicates a very good performance for the evaluated IgG ELISA tests, particularly Lionex, compared to other automated assays such as Roche Elecsys (sensitivity of 97.2%), Abbot (sensitivity of 92.7%) [32], and DiaSorin (sensitivity of 95.0%) [33]. Previous studies reported slightly better IgG sensitivity results for EDI and DiaPro; 76.1% vs. 82.7% for EDI and 53.5% vs. 92.2% for DiaPro [34]. NovaLisa showed comparable IgG sensitivity to other studies, 81.5% vs. 89.7% after two weeks post symptoms onset, and 90.0% vs. 91.2% after one month [35]. The IgM sensitivity for NovaLisa was higher in our study after two weeks (54.2% vs. 30.8%), but it significantly dropped after this (12.8% after one month vs. 38.2% after three weeks) [35,36]. This could be because many samples (~67%) collected at this time-interval were from asymptomatic patients whose samples were collected between 30 and 98 days post positive SARS-CoV-2 RT–PCR test. In addition, to our knowledge, serum IgM antibodies against SARS-CoV-2 decrease rapidly, potentially accounting for the poor assay sensitivity obtained after one month [8,35,37,38].

Regardless of the time of collection, our data demonstrated higher sensitivities for IgM and IgG in symptomatic COVID-19 patients compared to the asymptomatic patients (Figure 2 and Figure 5). In addition, a strong correlation between the clinical classification of COVID-19 and the detected antibody signal was observed. This was consistent with other studies reporting a stronger humoral immune response in severe COVID-19 patients compared to non-severe cases [39,40]. In addition, it was suggested that higher IgM levels were associated with poor disease prognosis, which could explain the low sensitivity in asymptomatic patients [38]. Still, further analysis is needed to assess the performance of the assays in patients with different clinical classification (mild, severe, and critical) and disease outcome, along with the other independent factors [41].

Our data demonstrated a specificity of around 90% for all IgM ELISA kits, EDI achieving nearly a 100% specificity. Three of the evaluated IgG ELISA kits demonstrated specificities above 95% (EDI, NovaLisa, and Lionex), which is comparable to the above-mentioned automated analyzers, including Abbot (99.9%) and Roche Elecsys (99.8%) [34]. Some of the evaluated ELISA kits showed cross-reactivity with samples seropositive for other respiratory viruses, including MERS, SARS-CoV, seasonal coronaviruses, influenza, and RSV, consistent with studies reporting cross-reactivity between SARS-CoV-2 and other human coronaviruses [36,42,43]. However, no consistent pattern of cross-reactivity was observed with the pre-pandemic samples by all evaluated ELISA kits. Hence, our results do not seem to suggest that the targeted antigen or type of assay (indirect vs. capture ELISA) significantly affects the specificity of the assays. Still, a larger sample cohort is needed to investigate these results further.

In addition, some studies pointed out that early antibody immune responses were targeted by the nucleocapsid protein (NP) [44,45], while another study revealed that immunoassays targeting the spike protein (SP) are more specific than the NP-based assays in detecting antibodies against SARS-CoV-2 [46]. However, this was not observed in the evaluated IgM ELISA kits, where two assays targeting the NP had slightly higher specificities (EDI and NovaLisa) compared to one assay targeting the SP (Lionex) (Table 3). Further, initial studies have suggested that IgM antibodies against SARS-Cov-2 might appear earlier than IgG and that measuring both IgM and IgG could improve the diagnosis of SARS-Cov-2 infection [47,48]. However, the performance of the IgM assays was variable and showed low sensitivity, consistent with other studies [36,49]. Therefore, combining IgM and IgG serology testing may not provide much diagnostic value during the later stages of the disease, questioning the rationale for measuring IgM antibodies. In addition, the early appearance of anti-SARS-CoV-2 IgG antibodies is interesting to note as similar studies have reported detectable responses as early as 3–5 days post-infection [32,50,51]. Usually, early detection of specific IgG antibodies is expected in secondary immune responses when there is a memory to cross-reactive antigens from a previous infection with a coronavirus [52,53]. A phenomenon known as the “original antigenic syndrome” has been suggested to explain the underlying immune response in SARS-CoV-2, where previous infections influence the response to future virus encounters [50]. The early IgG response is possibly a memory response from earlier coronavirus infection, producing cross-reactive antibodies that do not have neutralizing potency [52]. This was observed in several infections with closely related viruses, where some caused antibody-dependent enhancement (ADE) disease, leading to a worse clinical course [52,54]. In addition, this could explain why the symptomatic patients had a higher IgG detection rate than the asymptomatic patients.

Intriguingly, among the 291 samples collected from the RT-PCR-positive patients, we found 23 samples with no detectable anti-SARS-CoV-2 IgG antibodies by all ELISA kits. Among these cases, only seven samples showed detectable IgM antibodies by at least one of the four IgM ELISA kits. This could be due to several reasons, including false-positive RT–PCR test, reactive RT–PCR with high CT value, an early-stage infection, a transient antibody response, no production of an antibody response, or production of antibodies that are below the detection level of the assays [36,37,39,48]. Some studies have indicated similar findings where they failed to detect a SARS-CoV-2 antibody response in variable portions of their COVID-19 sample cohorts [54,55,56].

A strength of our study is that we used a diverse group of control samples to evaluate the cross-reactivity with other pathogens and other causes of false-positive results, including MERS-CoV, seasonal human coronaviruses, and other respiratory viruses. In addition, 291 samples from confirmed COVID-19 patients were used to assess the ELISA kits’ performance based on different time-intervals and disease classifications. Another strength is that we investigated the added value of measuring IgM antibodies along with IgG. One limitation of our study is that the onset time of illness was obtained from that patient medical records. This could have affected the precision of the time-intervals due to subjectivity in the perception of timing. In addition, most of the samples used from the asymptomatic patients were collected after one month of a positive PCR test. Hence, a more accurate evaluation of the IgM response in asymptomatic individuals could be obtained if more samples were available at the first two time-intervals.

## 5. Conclusions

In conclusion, this study showed a very good performance for three evaluated IgG assays (NovaLisa, AnshLabs, and Lionex), indicating that ELISA tests may have an essential diagnostic value in detecting antibodies against SARS-CoV-2 and developing epidemiological strategies for the COVID-19 pandemic. They will also be critical in determining the magnitude of antibody response needed for protection after SARS-CoV-2 vaccination, determining the antibody response’s durability, and distinguishing between natural and vaccine-induced immunity by differentiating binding antibodies from neutralizing antibodies elicited by the vaccines. Our study provided evidence that serological testing can be a powerful approach in achieving timely diagnosis and determining the level of humoral immunity in symptomatic and asymptomatic COVID-19 patients. The main expected use of antibody testing in the upcoming months is to confirm past infection, determine herd immunity, determine the durability of antibodies targeting different antigens, and test the currently approved vaccines’ efficacy.

## Figures and Tables

**Table 1:** Characteristics of the negative control group (*n* = 119) and coronavirus disease 2019 (COVID-19) patients (*n* = 291).

	Negative Controls			COVID-19 Patients		
	No (%)	Median (IQR ^2^)	Range	No (%)	Median (IQR ^2^)	Range
**Age (years)**						
All	119 (100)	36.0 (15.0)	20.0–69.0	291 (100)	43.0 (21.0)	12.0–91.0
10–30	23 (19.3)			52 (17.9)		
31–60	82 (68.9)			195 (67.0)		
60+	2 (1.7)			27 (9.3)		
**Gender**						
Female	57 (49.6)			33 (11.3)		
Male	59 (51.3)			242 (83.2)		
**Symptomatic**				147 (55.9)		
**Asymptomatic**				116 (44.1)		
**DPSO/DPD ^1^**						
≤14 days				119 (40.9)	8.0 (6.5)	0–14
14–30 days				55 (18.9)	19.5 (7.5)	14–30
>30 days				117 (40.2)	-	-

^1^ DPSO: days post symptoms onset, DPD: days post-diagnosis; ^2^ IQR: interquartile range.

**Table 2:** Characteristics of the evaluated immunoassays, including the recombinant antigen used, immunoglobulin (Ig) classes, and the reported sensitivity and specificity by the company.

Assay	Manufacturer	Detected Antibody	Principle of Detection	Antigen/Antibody Coating the Plate	Reported Sensitivity	Reported Specificity
EDI™ Novel Coronavirus COVID-19 ELISA Kit	Epitope Diagnostics, Inc.	IgM	Capture ELISA	Anti-human IgM specific capture antibody	45% (vs. RT-PCR ^1^)	100% (vs. PCR)
		IgG	Indirect ELISA	Recombinant full length nucleocapsid protein	100% (vs. RT-PCR)	100% (vs. PCR)
NovaLisa^®^ SARS-CoV-2 ELISA	NovaLisa Immundiagnostica GmbH	IgM	Indirect ELISA	Recombinant nucleocapsid antigen	0–30% (<11 days) 40% (≥12 days) (vs. RT-PCR)	100%
		IgG	Indirect ELISA	Recombinant nucleocapsid antigen	8–40% (<11 days) 100% (≥12 days) (vs. RT-PCR)	99.3%
AnshLabs SARS-CoV-2 ELISA	AnshLabs	IgM	Capture ELISA	Anti-human IgM specific capture antibody	100% (vs. CLIA ^2^) 40% (vs. RT-PCR)	98.5% (vs. CLIA) 100% (vs. PCR)
		IgG	Indirect ELISA	Recombinant nucleocapsid and spike antigens	95% (vs. CLIA) 83.6% (vs. RT-PCR)	98.3% (vs. CLIA) 91.3% (vs. PCR)
DiaPro COVID-19 ELISA	Diagnostic Bioprobes	IgG	Indirect ELISA	Recombinant nucleocapsid and spike antigens	≥98% (vs. RT-PCR)	≥98%
Lionex COVID-19 ELISA	Lionex Diagnostics and Therapeutics	IgM	Indirect ELISA	Recombinant S1 antigen	62.5% (vs. RT-PCR)	97.9%
		IgG	Indirect ELISA	Recombinant S1 antigen	>84% (vs. RT-PCR)	99.35%

^1^ RT–PCR: real-time polymerase chain reaction. ^2^ CLIA: chemiluminescent immunoassay.

**Table 3:** The specificity of the evaluated IgG and IgM ELISA tests according to the negative control subgroups (*n* = 119).

Control Subgroup	No. of Samples	IgG ELISA					IgM ELISA			
		EDI	NovaLisa	AnshLabs	DiaPro	Lionex	EDI	NovaLisa	AnshLabs	Lionex
Other coronaviruses (SARS-CoV, MERS-CoV, HCoV-229E, NL63, OC43, and HKU1)	20	19/20 (95.0%)	17/20 (85.0%)	17/20 (85.0%)	19/20 (95.0%)	20/20 (100%)	20/20 (100%)	20/20 (100%)	17/20 (85.0%)	15/20 (75.0%)
Non-CoV respiratory viruses (H1N1 influenza and RSV)	28	28/28 (100%)	19/28 (67.9%)	14/28 (50.0%)	26/28 (92.9%)	27/28 (96.4%)	28/28 (100%)	25/28 (89.3%)	27/28 (96.4%)	26/28 (92.9%)
Non-respiratory viruses (HEV, HGV, HCV, HBV, DENV, WNV, CHIKV, B19, HSV-1, HSV-2, EBV, HHV-6, and HHV-8)	65	64/65 (98.5%)	58/65 (89.2%)	54/65 (83.1%)	64/65 (98.5%)	63/65 (96.9%)	64/65 (98.5%)	62/65 (95.4%)	62/65 (95.4%)	59/65 (90.8%)
Antinuclear antibodies (ANAs)	6	6/6 (100%)	6/6 (100%)	5/6 (83.3%)	6/6 (100%)	6/6 (100%)	6/6 (100%)	6/6 (100%)	6/6 (100%)	5/6 (83.3%)
Overall Specificity	119	98.3% (117/119:96.0–100)	96.6% (115/119:93.4–99.9)	84.0% (100/119:77.5–90.6)	75.6% (90/119:67.9–83.3)	97.5% (116/119:94.7–100)	99.2% (118/119:97.5–100)	89.1% (106/119:83.5–94.7)	95.0% (113/119:91.0–98.9)	88.2% (105/119:82.4–92.0)

MERS: Middle East respiratory syndrome coronavirus, SARS-CoV: severe acute respiratory syndrome coronavirus, RSV: respiratory syncytial virus, HSV-1: herpes simplex virus 1, HSV-2 herpes simplex virus 2, HHV-6: human herpesvirus-6, HHV-8: human herpesvirus-8, EBV: Epstein–Barr virus, HBV: hepatitis B virus, HCV: hepatitis C virus, HEV: hepatitis E virus, HGV: hepatitis G virus, B19: parvovirus B19, WNV: West Nile virus.

**Table 4:** Characteristics of seronegative samples by all IgG ELISA kits.

Sample No.	Days between Sample Collection and Symptoms Onset/Diagnosis	Hospitalized/Non-Hospitalized	Disease Status	Disease Severity
1	≤14 days	Hospitalized	Symptomatic	Mild
2	≤14 days	Hospitalized	Symptomatic	Mild
3	≤14 days	Hospitalized	Symptomatic	Mild
4	≤14 days	Hospitalized	Symptomatic	Critical
5	≤14 days	Hospitalized	Symptomatic	Mild
6	≤14 days	Hospitalized	Symptomatic	Severe
7	≤14 days	Hospitalized	Symptomatic	Critical
8	≤14 days	Hospitalized	Symptomatic	Critical
9	≤14 days	Non-hospitalized	Asymptomatic	-
10	≤14 days	Non-hospitalized	Asymptomatic	-
11	≤14 days	Non-hospitalized	Asymptomatic	-
12	≤14 days	Non-hospitalized	Asymptomatic	-
13	≤14 days	Non-hospitalized	Asymptomatic	-
14	≤14 days	Non-hospitalized	Asymptomatic	-
15	≤14 days	Non-hospitalized	Asymptomatic	-
16	≤14 days	Non-hospitalized	Asymptomatic	-
17	≤14 days	Non-hospitalized	Asymptomatic	-
18	14–30 days	Hospitalized	Symptomatic	Critical
19	14–30 days	Non-hospitalized	Symptomatic	Severe
20	14–30 days	Non-hospitalized	Asymptomatic	-
21	>30 days	Non-hospitalized	Asymptomatic	Severe
22	>30 days	Non-hospitalized	Symptomatic	Mild
23	>30 days	Non-hospitalized	Asymptomatic	-

## Data Availability

Not applicable.

## Supplementary Materials

The following are available online at https://www.mdpi.com/2076-0817/10/2/161/s1, Table S1: The diagnostic assessment of IgG and IgM ELISA tests with RT-PCR at the three-time intervals (≤14, 14–30, >30 days) and in symptomatic/asymptomatic COVID-19 patients (*n* = 291).; Figure S1: Assays sensitivity according to time of sampling after symptoms onset or positive SARS-CoV-2 RT-PCR test from only symptomatic COVID-19 patients. Chi-square test was used to detect the presence of a statistically significant difference in the sensitivity between the time intervals in each assay.
