# Supplementary material for: Evaluation of Antibody Response in Symptomatic and Asymptomatic COVID-19 Patients and Diagnostic Assessment of New IgM/IgG ELISA Kits
Source: Pathogens. 2021 Feb 3;10(2):161. doi: 10.3390/pathogens10020161 (PMC7913500; doi:10.3390/pathogens10020161)

## Supplementary Materials

**Table S1.** The diagnostic assessment of IgG and IgM ELISA tests with RT-PCR at the three-time intervals (≤14, 14-30, >30 days) and in symptomatic/asymptomatic COVID-19 patients (n=291).

| **Diagnostic assessment** | **IgG ELISA** | | | | | **IgM ELISA** | | | |
|---|---|---|---|---|---|---|---|---|---|
| | **EDI** | **NovaLisa** | **AnshLabs** | **DiaPro** | **Lionex** | **EDI** | **NovaLisa** | **AnshLabs** | **Lionex** |
| Sensitivity **≤14** DPSO/DPD[1] | **49.6%** (59/119, 40.6-58.6) | **61.0%** (61/100, 51.4-70.6) | **78.2%** (93/119, 70.7-85.6) | **48.7%** (58/119, 39.8-57.7) | **58.6%** (58/99, 48.9-68.3) | **48.7%** (58/119, 39.8-57.7) | **46.2%** (55/119, 37.3-55.2) | **53.8%** (64/119, 44.8-62.8) | **66.4%** (79/119, 57.9-74.9) |
| Sensitivity **14-30** DPSO/DPD[1] | **61.8%** (34/55, 49.0-74.7) | **81.5%** (44/54, 71.1-91.8) | **83.6%** (46/55, 73.9-93.4) | **60.0%** (33/55, 47.1-72.9) | **81.5%** (44/54, 71.1-91.8) | **34.5%** (20/55, 22.0-47.1) | **29.1%** (16/55, 17.1-41.1) | **38.2%** (21/55, 25.3-51.0) | **61.8%** (34/55, 49.0-74.7) |
| Sensitivity **>30** DPSO/DPD[1] | **76.1%** (89/117, 68.3-83.8) | **90.0%** (99/110, 84.4-95.6 | **95.7%** (112/117, 92.1-99.4) | **53.5%** (38/71, 41.9-65.1) | **96.6%** (113/117, 93.3-99.9) | **10.3%** (12/117, 4.8-15.8) | **12.8%** (15/117, 6.8-18.9) | **6.8%** (8/117, 2.3-11.4) | **47.9%** (56/117, 38.8-56.9) |
| Sensitivity in **symptomatic** COVID-19 patients | **71.4%** (104/147, 64.1-78.7) | **84.1%** (111/132, 77.9-90.3) | **89.1%** (131/147, 84.1-94.2) | **67.3%** (99/147, 59.8-74.9) | **84.1%** (111/132, 77.9-90.3) | **52.4%** (77/147, 44.3-60.5) | **45.6%** (67/147, 37.5-53.6) | **57.1%** (84/147, 49.1-65.1) | **75.5%** (111/147, 68.6-82.5) |
| Sensitivity in **asymptomatic** COVID-19 patients | **56.0%** (65/116, 47.0-65.1) | **67.9%** (74/109, 59.1-76.7) | **86.2%** (100/116, 79.9-92.5) | **24.3%** (18/70, 14.2-34.3) | **71.6%** (83/116, 63.3-79.8) | **7.8%** (9/116, 2.9-12.6) | **13.8%** (16/116, 7.5-20.1) | **3.4%** (4/116, 0.13-6.8) | **39.7%** (46/116, 30.8-48.6) |
| **Overall** sensitivity | **62.5%** (182/291, 57.0-68.1) | **77.6%** (204/263, 72.5-82.6) | **86.3%** (251/291, 82.3-90.2) | **52.7%** (129/245, 46.4-58.9) | **80.0%** (216/270, 75.2-84.8) | **30.6%** (89/291, 25.3-35.9) | **29.6%** (86/291, 24.3-34.8) | **31.6%** (92/291, 26.3-37.0) | **58.4%** (170/291, 52.8-64.1) |
| Overall agreement with RT-PCR | **72.9%** (68.6-77.2) | **83.5%** (79.8-87.2) | **85.6%** (82.2-89.0) | **60.2%** (55.1-65.2) | **85.3%** (81.8-88.9) | **50.5%** (45.6-55.3) | **46.8%** (42.0-51.7) | **50.0%** (45.2-54.8) | **67.1%** (62.5-71.6) |
| Positive predictive value | **98.9%** (97.9-99.9) | **98.1%** (96.7-99.5) | **93.0%** (90.5-95.4) | **81.6%** (77.6-85.6) | **98.6%** (97.5-99.8) | **98.9%** (97.8-99.9) | **86.9%** (83.6-90.1) | **93.9%** (91.6-96.2) | **92.4%** (89.8-94.9) |
| Negative predictive value | **51.8%** (46.9-56.6) | **66.1%** (61.3-70.8) | **71.4%** (67.1-75.8) | **43.7%** (38.6-48.8) | **68.2%** (63.6-72.9%) | **36.9%** (32.2-41.5) | **34.1%** (29.5-38.7) | **36.2%** (31.6-40.9) | **46.5%** (41.6-51.3) |

[1]DPSO: Days post symptoms onset, DPD: days post diagnosis

**Figure S1.** Assays sensitivity according to time of sampling after symptoms onset or positive SARS-CoV-2 RT-PCR test from only symptomatic COVID-19 patients. Chi-square test was used to detect the presence of a statistically significant difference in the sensitivity between the time intervals in each assay.

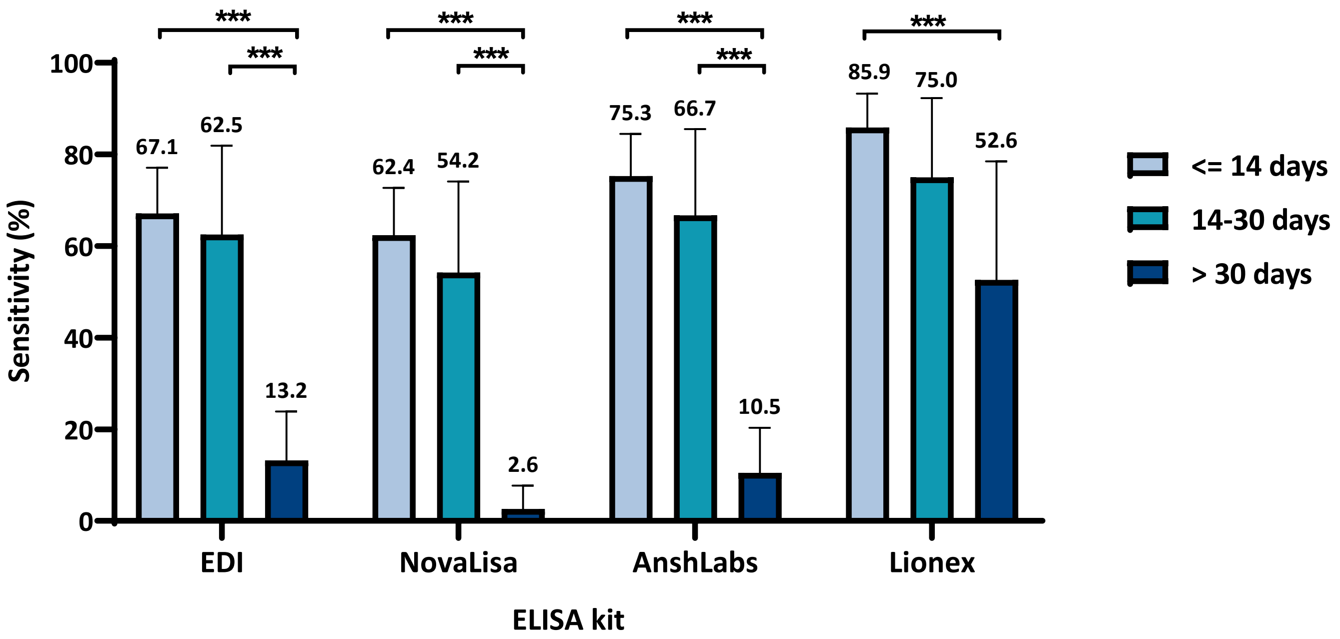

Supplement: Supplementary file 1 [file pathogens-10-00161-s001.pdf]
